# Multi-Omics Characterization of a Glycerolipid Metabolism-Related Gene Enrichment Score in Colon Cancer

**DOI:** 10.3389/fonc.2022.881953

**Published:** 2022-05-05

**Authors:** Zhiyu Wang, Zhuoqi Zhang, Ke Zhang, Qiaoxia Zhou, Sidong Chen, Hao Zheng, Guoqiang Wang, Shangli Cai, Fujing Wang, Shenglong Li

**Affiliations:** ^1^ Department of Medical Oncology, Hebei Key Laboratory of Cancer Radiotherapy and Chemotherapy, Affiliated Hospital of Hebei University, Baoding, China; ^2^ Department of Gastrointestinal Surgery, Affiliated Hospital of Hebei University, Baoding, China; ^3^ General Surgery Department, The Second Affiliated Hospital of Harbin Medical University, Harbin, China; ^4^ Medical Department, Burning Rock Biotech, Guangzhou, China

**Keywords:** glycerolipid metabolism, colon cancer, prognostic signature, overall survival, multi-omics characterization

## Abstract

**Background:**

Glycerolipid metabolism is involved in the genesis and progression of colon cancer. The current study aims at exploring the prognostic value and potential molecular mechanism of glycerolipid metabolism-related genes in colon cancer from the perspective of multi-omics.

**Methods:**

Clinical information and mRNA expression data of patients with colon cancer were obtained from The Cancer Genome Atlas (TCGA) and Gene Expression Omnibus (GEO) databases. Single-sample gene set enrichment analysis (ssGSEA) was applied to calculate the glycerolipid metabolism-related gene enrichment score (GLMS). Univariable and multivariable Cox regression analyses were used to study the prognostic value of GLMS in TCGA-COAD and GSE39582 cohorts. The molecular mechanism of the prognostic factor was investigated *via* immune cell infiltration estimation and correlation analysis of cancer hallmark pathways. Single-cell transcriptomic dataset GSE146771 was used to identify the cell populations which glycerolipid metabolism targeted on.

**Results:**

The GLMS was found to be associated with tumor location and consensus molecular types (CMSs) of colon cancer in TCGA-COAD cohort (*P* < 0.05). Patients in the low-GLMS group exhibited poorer overall survival (OS) in TCGA cohort (*P* = 0.03; HR, 0.63; 95% CI, 0.42–0.94), which was further validated in the GSE39582 dataset (*P* < 0.001; HR, 0.57; 95% CI, 0.43–0.76). The association between the GLMS and OS remained significant in the multivariable analysis (TCGA cohort: *P* = 0.04; HR, 0.64; 95% CI, 0.42–0.98; GSE39582 cohort: *P* < 0.001; HR, 0.60; 95% CI, 0.45–0.80). The GLMS was positively correlated with cancer hallmark pathways including bile acid metabolism, xenobiotic metabolism, and peroxisome and negatively correlated with pathways such as interferon gamma response, allograft rejection, apoptosis, and inflammatory response (*P* < 0.05). Increased immune infiltration and upregulated expression of immune checkpoints were observed in patients with lower GLMS (*P* < 0.05). Single-cell datasets verified the different distribution of GLMS in cell subsets, with significant enrichment of GLMS in malignant cells and Tprolif cells.

**Conclusion:**

We demonstrated that GLMS was a potential independent prognostic factor for colon cancer. The GLMS was also correlated with several cancer hallmark pathways, as well as immune microenvironment.

## Introduction

Colon cancer is the third most commonly diagnosed malignant tumor all over the world, accounting for almost 10% of all the cancer-related deaths in 2020 (1). Despite the rapid development in advanced surgical techniques and therapeutic strategies, the 5-year survival rate of late-stage colon cancer remains less than 30% due to metastasis and post-operation recurrence (2). The prognosis of colon cancer varies among different patients due to its high heterogeneity (3), since multifarious risk factors and multiple gene alterations are involved in its pathogenesis and progression, leading to distinct clinical pathological features and diverse responses to treatments (4). Nowadays, the establishment of public databases such as The Cancer Genome Atlas (TCGA) project and Gene Expression Omnibus (GEO) has enabled numerous studies exploring prognostic and predictive gene markers for colon cancer management; however, there is still a long way off identifying universally applied robust models.

Aberrant activation of lipid metabolism is an important hallmark for cancer cells (5), resulting in alterations of the lipid composition profile in different tumor tissues including lung (6), breast (7), and liver cancers (5). The lipid synthesis provides essential substrate for energy metabolism and ingredients for cell membrane construction during the proliferation process, suggesting an indispensable role of lipid metabolism pathways in the genesis and development of cancer. A subsequent study verified an upregulated expression of the key enzymes for lipid synthesis in colon cancer cells (8), followed by attempts of utilizing lipid synthesis inhibitors in colon cancer treatment (9), which suggested the vital role of lipid metabolism in colon cancer development. Among the various metabolites, triacylglycerol has been identified as a prognostic biomarker for colon cancer (10), indicating a possible contribution of glycerolipid metabolism in colon cancer progression. Therefore, genes related to glycerolipid metabolism may serve as potential prognostic markers of colon cancer, and a better understanding of cancer-related genes involved in glycerolipid metabolism may provide a reference for the selection of treatment strategies in colon cancer patients. However, relevant studies regarding glycerolipid metabolism and colon cancer development are still limited.

Thus, we conducted this study based on public databases including TCGA and GEO, proposing a prognostic model of colon cancer based on glycerolipid metabolism genes, and further explored the molecular mechanism of the glycerolipid metabolism-related genes involved in the development of colon cancer from the perspective of multi-omics.

## Methods and Materials

### Data Acquisition

Three datasets were involved in this study. Clinical information and survival data of COAD patients from The Cancer Genome Atlas (TCGA-COAD) were downloaded from the UCSC XENA database (https://xenabrowser.net/), as well as the gene expression matrix (HTSeq-FPKM), somatic mutation, copy number variation (CNV, gene-level), and methylation (Illumina Human Methylation 450) sequencing results. Clinical information and survival data of colon cancer patients in the GSE39582 dataset were retrieved from the Gene Expression Omnibus (GEO) dataset (http://www.ncbi.nlm.nih.gov/geo). Microarray RAW “CEL” data files of colon cancer patients in the GSE39582 dataset were also downloaded, and normalization was conducted through the Robust Multiarray Average (RMA) method in R package “Affy.” A single-cell transcriptomic dataset of COAD patients in GSE146771 (Smart-seq2) was also obtained from the GEO database. The KEGG_GLYCEROLIPID_METABOLISM gene set and 50 cancer hallmark gene sets were downloaded from the Molecular Signature Database v. 7.4 (MSigDB). A total of 49 glycerolipid metabolism-related genes were obtained from the KEGG_GLYCEROLIPID_METABOLISM pathway.

### Clinical Dataset Preprocessing

The following preprocessing steps were applied to both TCGA-COAD and GSE39582 datasets. (1) Patients who had no clinical information were excluded from the datasets. (2) Patients whose survival time was 0 or survival status was unknown were excluded from the analysis. (3) The type of gene ID in the gene expression matrix of both datasets was synchronized to Gene Symbol. When multiple gene IDs were synchronized to one Gene Symbol, the median value was selected to represent the expression level.

### Multi-Omics Characterization of Glycerolipid Metabolism in Colon Cancer

The 49 glycerolipid metabolism-related genes were mapped to the gene expression matrix and gene methylation matrix. The gene expression level and methylation level of these genes were compared between tumor and normal tissues by the Mann–Whitney test, and *P*-value <0.05 was considered significant. The CNV amplification and deletion frequency, as well as the somatic single-nucleotide variation, of the glycerolipid metabolism-related genes were calculated from relative sequence results of tumor samples.

### Single-Sample Gene Set Enrichment Analysis

Single-sample gene set enrichment analysis (ssGSEA) is an unsupervised single-sample gene set enrichment analysis method (11). ssGSEA calculates the enrichment score of each sample by the cumulative distribution function difference between gene expression ranks inside the settled gene set and outside. The enrichment score was further normalized by the range of total gene expression matrix for all genes and samples (11). The ssGSEA analysis method included in R package “GSVA” was used to calculate the KEGG_GLYCEROLIPID_METABOLISM gene set enrichment score (GLMS) based on the gene expression matrix of TCGA-COAD and GSE39582 (12). Samples were categorized into high- and low-GLMS groups based on the median of GLMS for both TCGA-COAD and GSE39582 datasets.

### Association Between Clinical Characteristics and GLMS

The consensus molecular subtypes (CMSs) of TCGA-COAD datasets were estimated *via* the R package “CMScaller” (13). The clinical characteristics of TCGA-COAD datasets such as age, sex, BMI (classified into high and low groups based on median), stage, microsatellite instability, venous invasion, and tumor location were included for assessing the association between the GLMS and clinical characteristics, together with the CMS subtypes.

### Pathway Correlation Analysis

The ssGSEA analysis method was also applied to calculate the 50-cancer hallmark gene set enrichment score based on the gene expression matrix of TCGA-COAD dataset. Spearman correlation analysis was used to quantify the correlation between the GLMS and 50 cancer hallmark gene sets based on their ssGSEA scores of TCGA-COAD samples. The correlation between every single glycerolipid metabolism-related gene in the KEGG gene set and 50 cancer hallmark gene sets was also calculated *via* R package “WGCNA” corAndPvalue function based on gene expression and the hallmark ssGSEA score (14).

### Immune Microenvironment Analysis

The proportions of 64 cell types in the tumor microenvironment of TCGA-COAD tumor samples were estimated by R package “xCell” (15). The expressions of 14 immune checkpoints were compared between high- and low-GLMS groups including CD274, CD276, CD40, CTLA4, HAVCR2, ICOS, IDO1, LAG3, PDCD1, TIGIT, TNFRSF18, TNFRSF4, TNFRSF9, and VTCN1.

### Potential Drug Sensitivity Analysis

The pRRophetic algorithm was originally developed for drug response prediction based on gene expression microarray data and the half-maximal inhibitory concentration (IC50) of 138-drug response data in almost 700 cell lines from the Cancer Genome Project (CGP) database (16, 17). The drug sensitivity of those drugs in TCGA-COAD dataset, as indicated by IC50 prediction, was calculated based on the R package “pRRophetic” and gene expression matrix of these samples. The difference of estimated drug sensitivity between high- and low-GLMS groups was evaluated for every drug *via* the Wilcoxon test, and *P*-value <0.05 was considered significant. Subsequently, the correlation between every single gene in the KEGG_GLYCORLIPID_METABOLISM gene set and drug sensitivity was calculated *via* R package “WGCNA” corAndPvalue function based on expression and IC50 (14). Furthermore, potential drug response-related genes were selected according to *P*-value and correlation coefficient (*P*-value< 0.05, |cor| >0.5).

### Single-Cell Transcriptomic Analysis

GLMS of single-cell transcriptomic dataset GSE146771 was calculated by the ssGSEA method as well. The cell annotation information corresponding to this dataset was downloaded from the Tumor Immune Single-cell Hub (TISCH) database (http://tisch.comp-genomics.org). The GLMS of every single cell was shown by uniform manifold approximation and projection (UMAP) plots at two resolutions: “Celltype malignancy” and “Celltype major lineage.” The Kruskal–Wallis test was applied to evaluate whether GLMS varied among different cell types, and *P*-value <0.05 was considered significant. Finally, the cell subpopulations on which glycerolipid metabolism affected were estimated by the log2FoldChange (log2FC) value of each cell types *via* “one vs. rest” calculation.

### Statistical Analysis

Data preprocessing and R package analyses were completed in R (version 4.1). Statistical analysis and figures were completed in GraphPad Prism (version 9). Continuous variables were displayed as median (range), and categorical variables were displayed as number (percentage). The Wilcoxon test was applied to calculate the significance of the difference in continuous variables between two groups, and the chi-square test or Fisher’s exact test was used to calculate the significance of the difference in categorical variables between groups. Kaplan–Meier analysis was performed to estimate the overall survival (OS) rates, and the difference of survival curves between high- and low-GLMS groups was compared by the log-rank test through R package “survival.” Univariable and multivariable Cox proportional hazards regression analyses were conducted *via* R package “survival” coxph function to determine whether the GLMS could be an independent prognostic factor for colon cancer. Statistical significance was considered as *P*-value <0.05.

## Results

### Epigenetic and Genomic Characteristics of Glycerolipid Metabolism-Related Genes in Colon Cancer

**Figure 1:**
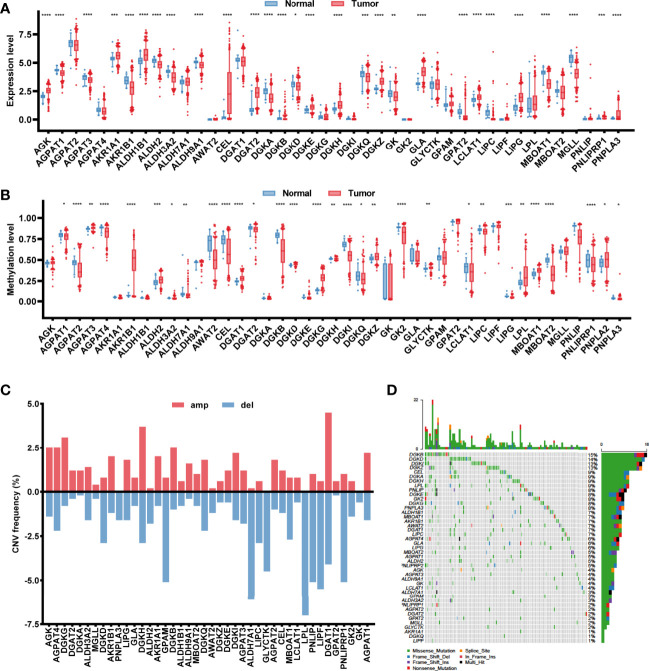
Characterization of the glycerolipid metabolism genes in colon cancer patients. **(A, B)** The mRNA expression **(A)** and methylation **(B)** of the glycerolipid metabolism-related genes between tumor and normal tissues from TCGA database. **(C, D)** Copy number variation (CNV) frequency showing gene amplification (amp) and deletion (del) **(C)** and mutation oncoprint **(D)** of 42 glycerolipid metabolism-related genes in 430 tumor tissues from TCGA-COAD cohorts. *P < 0.05; **P < 0.01; ***P < 0.001; and ****P < 0.0001 by the Mann–Whitney test.

Based on the 430 cancer samples and 39 normal samples in TCGA-COAD dataset, the expression levels of 43 glycerolipid metabolism-related genes were available out of 49 genes from the KEGG_GLYCEROLIPID_METABOLISM gene set, while the methylation information was provided for 42 genes. The baseline characteristics of the samples in TCGA-COAD dataset are described in **Supplementary Table**. Differences in expression and methylation levels were compared between tumor and normal tissues (**Figure 1A**), which identified nine genes with a negative correlation between expression and methylation levels, including CEL, DGAT2, LCLAT1, and LIPG (hypomethylated and upregulated in tumor tissues), and AGPAT3, AKR1B1, ALDH2, DGKD, and MBOAT1 (hypermethylated and downregulated in tumor tissues), indicating an inhibitory function of methylation in the mRNA expression of those genes. However, the methylation of six genes was positively correlated with expression levels, including DGKH and DGKZ (hypermethylated and upregulated in tumors), and AGPAT1, DGKB, DGKQ, and LIPC (hypomethylated and downregulated in tumors), suggesting the potential regulatory mechanisms of mRNA expression in those genes beyond methylation. The copy number variations and gene mutations were also analyzed based on cancer samples. Copy number amplification appeared in AGPAT1, AKR1B1, DGKB, DGAT2, and DGKH, while AGPAT3, ALDH2, DGKD, MBOAT1, and LIPC exhibited significant copy number deletions (**Figure 1**). A total of 120 (27.9%) tumor samples harbored mutations in glycerolipid metabolism-related genes, with two genes identified as high frequently mutated, including DGKB (15%) and DGKD (14%) (**Figure 1**).

### Glycerolipid Metabolism Score as a Prognostic Indicator for Colon Cancer

**Figure 2:**
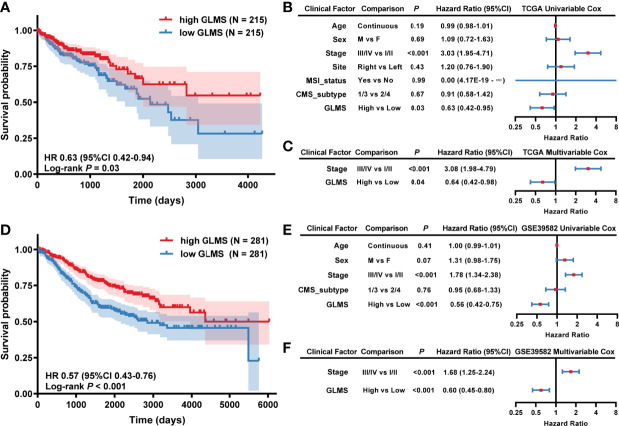
Association between glycerolipid metabolism score (GLMS) and survival in colon cancer patients. **(A)** Kaplan–Meier survival curves of overall survival comparing the high- and low-GLMS groups in TCGA-COAD dataset, which showed improved median overall survival (OS) in the high-GLMS group compared with the low-GLMS group (not reached vs. 2,134 days). **(B, C)** The forest plot of the univariable **(B)** and multivariable **(C)** Cox regression analysis in TCGA-COAD cohort. **(D)** Kaplan–Meier survival curves of overall survival comparing the high- and low-GLMS groups in the GSE39582 dataset, which showed improved median overall survival (OS) in the high-GLMS group compared with the low-GLMS group (not reached vs. 2,850 days). **(E, F)** The forest plot of the univariable **(E)** and multivariable **(F)** Cox regression analysis in the GSE39582 cohort. HR, hazard ratio; 95% CI, 95% confidence interval; CMS, consensus molecular subtype; MSI, microsatellite instability.

Each sample in TCGA-COAD dataset was assigned with a glycerolipid metabolism-related gene enrichment score (GLMS) according to the single-sample gene set enrichment analysis (ssGSEA) algorithm and divided into high- and low-GLMS groups by the cutoff of the median value. We explored whether the GLMS could predict the prognosis of colon cancer in TCGA-COAD dataset. Kaplan–Meier analysis showed that the patients with higher GLMS exhibited improved overall survival (OS) compared with those with lower GLMS (log-rank *P* = 0.03; HR, 0.63; 95% CI, 0.42–0.94, **Figure 2**). Univariable analysis was also conducted in other clinical factors, including age, sex, stage, tumor location, MSI status, and CMS, among which only clinical stage and GLMS exhibited statistical significance (*P* < 0.05, **Figure 2**). These clinical factors along with GLMS were included in the multivariable Cox regression, and the association between GLMS and OS remained significant (*P* = 0.04; HR, 0.64; 95% CI, 0.42–0.98, **Figure 2**), suggesting that the GLMS was a potential independent prognostic factor of colon cancer.

To further verify the robustness of the GLMS in predicting the prognosis of colon cancer, another dataset (GSE39582) was used as a validation cohort. A total of 562 tumor samples were included in the GSE39582 dataset, with the baselined characteristics described in **Supplementary Table**. With the same algorithm of GLMS, patients in the high-GLMS group also had a better OS (log-rank *P* < 0.001; HR, 0.57, 95% CI, 0.43–0.76, **Figure 2**). Similar results were also observed in the univariable (**Figure 2**) and multivariable Cox regression (**Figure 2**). Univariable analysis has identified tumor stage and GLMS that were associated with OS (**Figure 2**). The GLMS was further confirmed to be an independent prognostic factor of OS in the multivariable analysis (*P* < 0.001; HR, 0.60; 95% CI, 0.45–0.80, **Figure 2**). Taken together, these results suggested that the GLMS was an independent prognostic factor for colon cancer.

### Association Between GLMS and Clinical Features

**Figure 3:**
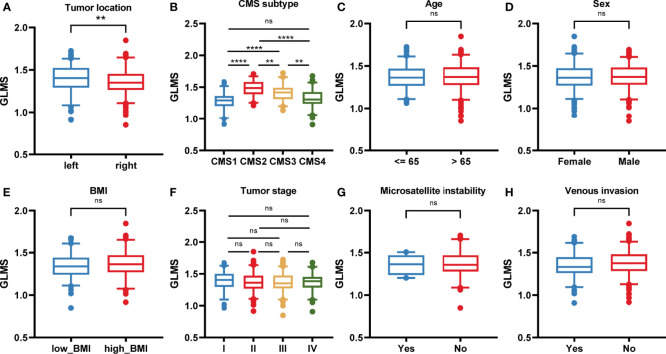
The association between the glycerolipid metabolism score and clinical features. **(A–H)** The glycerolipid metabolism score (GLMS) distribution in patients with different clinicopathologic features including tumor location **(A)**, and consensus molecular subtype (CMS) **(B)**, age **(C)**, sex **(D)**, body mass index (BMI) **(E)**, tumor stage **(F)**, microsatellite instability status **(G)**, and venous invasion status **(H)**. ns (non-significant) *P* > 0.05, **P < 0.01 and ****P < 0.0001 by the Wilcoxon test.

The potential association between GLMS and clinical features was further analyzed including age, sex, body mass index (BMI), tumor stage by American Joint Committee on Cancer (AJCC) TNM Classification, microsatellite status, vascular invasion status, tumor location, and consensus molecular subtypes (CMS) (**Figure 3**). Results showed that left colonic carcinomas exhibited significantly higher GLMS compared with right ones (*P* = 0.008, **Figure 3**), suggesting the capacity of the GLMS in differentiating the tumor location. Besides, CMS subtypes among tumors have revealed significant differences in GLMS distribution (*P* < 0.05, **Figure 3**), while patients in different groups by age, sex, BMI, tumor stage, microsatellite instability status, and venous invasion status were observed to have a similar distribution of the GLMS (**Figure 3C**).

### Underlying Mechanism of Glycerolipid Metabolism

**Figure 4:**
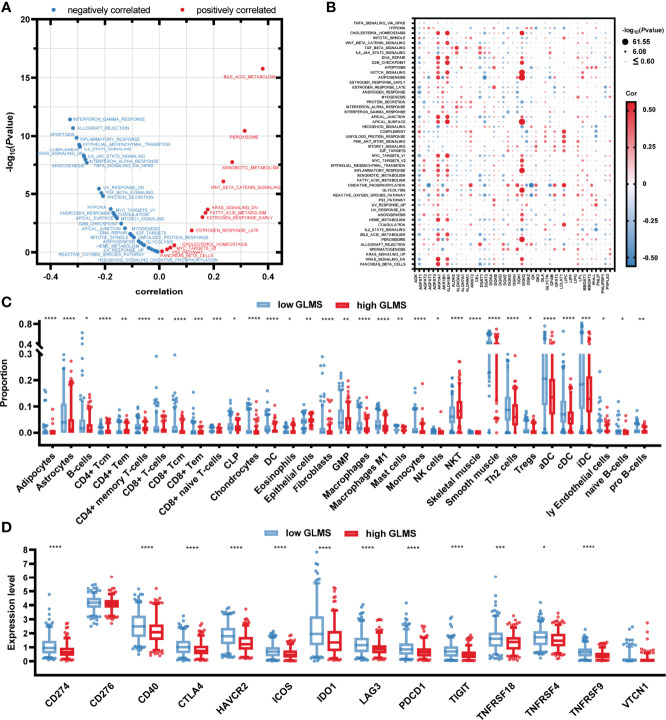
Potential molecular mechanisms of glycerolipid metabolism. **(A)** Correlation between glycerolipid metabolism score (GLMS) and ssGSEA enrichment scores of 50 cancer hallmark pathways in TCGA-COAD cohort. **(B)** Correlation between the expression level of 42 glycerolipid metabolism-related genes and ssGSEA enrichment scores of 50 cancer hallmark pathways. **(C)** The abundance of each tumor microenvironment (TME)-infiltrating cell between high- and low-GLMS groups in TCGA-COAD cohort. **(D)** The difference of 14-immune checkpoint mRNA expression between high- and low-GLMS groups in TCGA-COAD cohort. *P < 0.05, **P < 0.01, ***P < 0.001 and ****P < 0.0001 by the Mann–Whitney test.

To explore the potential pathways glycerolipid metabolism was involved in, the correlation between the GLMS and cancer hallmarks was analyzed. A significant positive association was observed between GLMS and pathways including bile acid metabolism, xenobiotic metabolism, and peroxisome, while pathways such as interferon gamma response, allograft rejection, apoptosis, and inflammatory response were found to be negatively correlated with the GLMS (**Figure 4**). The 42 genes involved in the GLMS were analyzed as well. Samples with an upregulated expression of AGPAT4, AKR1B1, and DGKI showed positive enrichment in pathways including cholesterol homeostasis, bile acid metabolism, inflammatory response, and NOTCH signaling (**Figure 4**), while expressions of AGPAT4, AKR1B1, DGKI, and MGLL were negatively associated with pathways such as protein secretion and IL2-STAT5 signaling (**Figure 4**), suggesting the core genes and pathways involved in glycerolipid metabolism.

The immune microenvironment was revealed by utilizing R package “xCell” to estimate the infiltration of the proportion of different immune cells, which was further compared between high- and low-GLMS groups. As presented in **Figure 4**, a total of 33 immune cells or stromal cells exhibited a significant infiltration difference between samples with high- and low-GLMS. Most of the immune-promoting cells, including B cells, macrophages, activated dendritic cells (aDC), classical dendritic cells (cDC), and immature dendritic cells (iDC), accounted for higher proportions in the low-GLMS group, while cells negatively regulating immune response such as NKT cells were significantly infiltrated in high-GLMS group samples.

Furthermore, the expression of 14 genes involved in immune checkpoints was compared between the two groups. As a result, a total of 12 immune checkpoint molecules were upregulated in low-GLMS group samples (**Figure 4**), suggesting a potential benefit of immune therapies in patients with low GLMS.

### Drug Sensitivity

**Figure 5:**
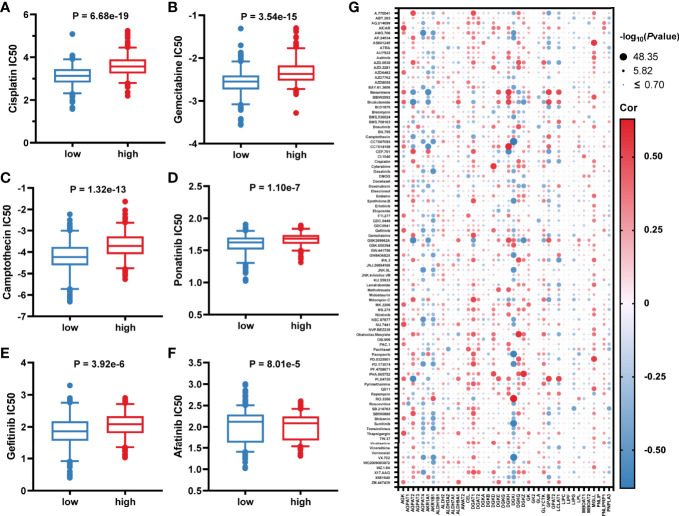
Association between glycerolipid metabolism score and drug sensitivity. **(A–F)** The correlation between glycerolipid metabolism scores (GLMS) of cell samples and estimated half-maximal inhibitory concentration (IC50) value of drugs including cisplatin **(A)**, gemcitabine **(B)**, camptothecin **(C)**, ponatinib **(D)**, gefitinib **(E)**, and afatinib **(F)**. **(G)** The correlation between glycerolipid metabolism-related genes and estimated IC50 value of 96 drugs.

Considering that lower GLMS was associated with poor prognosis, we conducted further analysis exploring the relationship between GLMS and drug sensitivity. Based on the Genomics of Drug Sensitivity in Cancer (GDSC) database, the half-maximal inhibitory concentration (IC50) information was acquired to predict the treatment response. Among the 96 drugs which exhibited a significant response difference between groups with low and high GLMS, those that were currently used in the treatment of colon cancer were paid extra attention. Low-GLMS group samples were observed to be more sensitive to chemotherapeutics including cisplatin (**Figure 5**), gemcitabine (**Figure 5**), and camptothecin (**Figure 5**), which had analogues of oxaliplatin, capecitabine, and irinotecan, respectively. Besides, ponatinib (**Figure 5**) as an anti-angiogenesis inhibitor targeting vascular endothelial growth factor receptors (VEGFRs) and epidermal growth factor receptor (EGFR) tyrosine kinase inhibitors (TKIs) including gefitinib (**Figure 5**) and afatinib (**Figure 5**) also exhibited significant increased sensitivity associated with low-GLMS group samples, suggesting potential benefits utilizing these therapies in patients with lower GLMS who had poorer survival.

In addition, further analysis also explored the association between IC50 of the drugs and 42-glycerolipid metabolism-related gene expression. According to *P*-value <0.05 and the absolute value of correlation >0.5, a total of 14 genes were identified including AKR1B1, DGKI, AGPAT2, GPAM, LCLAT, DGKZ, MGLL, DGKQ, AGPAT4, DGAT1, AKR1A1, DGKD, DGKH, and AGK (**Figure 5**), suggesting that these genes might play a crucial role in drug response.

### Origination of Glycerolipid Metabolic Disorders

**Figure 6:**
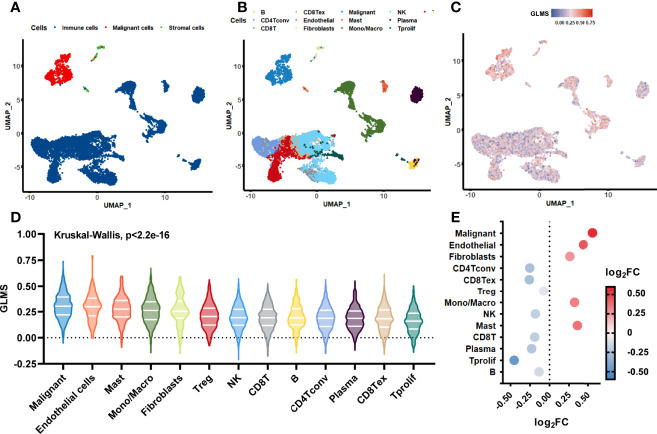
Glycerolipid metabolism score in single-cell datasets. **(A, B)** The distribution of the cell samples by uniform manifold approximation and projection (UMAP) presented in rough classification of “malignant-immune-stromal cells” **(A)** and detailed subsets **(B)**. **(C)** The UMAP distribution of the cells presented with glycerolipid metabolism scores (GLMS) of each sample. **(D)** Distribution difference of the GLMS in 13 subsets of cells with statistical analysis by the Kruskal–Wallis test. **(E)** GLMS enrichment by log2 fold change (log2FC) value in 13 subsets of cells.

The single-cell sequencing dataset GSE146771 was introduced in this current study to investigate the origination of glycerolipid metabolic disorders. Annotations of cell samples were obtained from the TISCH database to identify the subtype of the cells, which were presented by a rough classification of “malignant-immune-stromal cells” (**Figure 6**) and detailed subsets (**Figure 6**). Each cell sample was assigned with a GLMS according to the same ssGSEA algorithm (**Figure 6**). Results showed that the scores were generally higher in malignant cells and stromal cells, as well as in CD4Tconv, CD8Tex, and plasma cells when it came to the sophisticated categories. Further statistical analysis revealed a significant difference of the score distribution among various cell types (*P* < 2.2e-16) (**Figure 6**). Enrichment of the scores was analyzed based on the log2FC value of the scores in each cell subset, which showed significant enrichment in malignant cells and Tprolif cells (**Figure 6**), suggesting potential targets and originations of the glycerolipid metabolic disorders.

## Discussion

Over the past decade or so, researchers have been extensively working on cancer metabolism as it has been considered as one of the cancer hallmarks (18, 19). Lipid synthesis and metabolism are known to get involved in cell proliferation (19–22). Additionally, it is reported that lipidomic profiling is significantly different between tumor tissues and healthy tissues (5–7). However, even though colon cancer patients with obesity and metabolic dysfunction have worse survival (23), there is still debate on whether lipid metabolism and related genes contribute to the disease progression of colorectal cancer (CRC) patients (24–26).

Glycerolipid is one of the most important lipid molecules, but little is known about glycerolipid metabolism in colon cancer (27). To address these questions, we developed a prognostic model GLMS for colon cancer based on glycerolipid metabolism-related genes *via* the ssGSEA algorithm and proved that the GLMS was an independent clinical prognostic factor. We further investigated the molecular mechanism behind this model by evaluating the cross talk between GLMS and cancer hallmark pathways, as well as the immune microenvironment. Surprisingly, we observed that the glycerolipid metabolism pathway was highly correlated with several cancer hallmark pathways and the immune microenvironment. Last but not least, we identified that malignant and Tprolif cells were the most possible cell populations which glycerolipid metabolism targeted on.

Over the past few years, many prognostic models have been established based on gene signature or other biological molecules from different signaling pathways of colon cancer (28). A majority part of these prognostic models was based on immune-relevant signaling pathways. For example, Zhou et al. constructed a tumor microenvironment risk score (TMRS) with 100 tumor microenvironment-related genes based on the GEO database using the Lasso Cox regression model. This model was an effective tool for survival prediction of not only colon cancer but also gastric cancer (29). Xu et al. developed a prognostic model for colon cancer with 11 immune-related genes based on single-cell sequencing data (30). Intriguingly, one recent study identified that one single immune-related gene, CXCL11, could be an effective independent prognostic biomarker for colon cancer patients and upregulation of CXCL11 expression was correlated with a PD-L1 high expression level (31). For miRNA-constructed models, Zhang et al. used Lasso Cox regression to construct a classifier of 6 miRNAs which could predict recurrence in patients with stage II colon cancer and whether patients would benefit from postoperative adjuvant therapy (32).

Surprisingly, not many prognostic models based on lipid synthesis- and metabolism-related genes have been developed so far. Jiang et al. constructed a prognostic model of 8 lipid metabolism-related genes for colorectal cancer based on TCGA and GEO databases (33). A latest research investigated the significance of lipid metabolism for the prognostic of CRC based on multi-omics. In their research, a Cox regression analysis of lipid metabolites was utilized to identify the key lipid metabolites and construct the prognostic model. Furthermore, they distinguished the glycerophospholipid metabolism-related hub genes and developed a prognostic signature through Cox regression and LASSO regression analysis (34). Moreover, a research team developed and validated a robust prognostic model for colorectal cancer based on a lipid species molecule signature from lipidomics quantification analysis instead of gene or miRNA (10). More importantly, one major component of this robust CRC-specific lipid signature model was glycerolipid which gave us a hint that glycerolipid metabolism might be very important for colon cancer prognostic prediction. To date, our study provided the first colon cancer prognostic factor based on the glycerolipid metabolism-related gene enrichment score.

Pathway correlation analysis results suggested that glycerolipid metabolism was significantly positively correlated with bile acid metabolism pathway, peroxisome pathway, and xenobiotic metabolism pathway. In terms of the peroxisome pathway, peroxisome proliferator-activated receptor γ (PPARγ) is a nuclear receptor that regulates the expression of genes related to lipid metabolism and known to have anticancer activity (35). It is reported that tumor tissues of colon cancer patients expressing PPARγ have better prognosis, which supports our prognostic factor and correlation analysis (36). A high plasma bile acid level is reported to be positively correlated with colon cancer risk. This result indicated that a high load of bile acid due to inadequate metabolism might be promotive for colon cancer tumorigenesis and disease progression which was consistent with our analyses as well (37). On the other hand, analysis results indicated that the interferon-γ (IFN-γ) response pathway was negatively correlated with glycerolipid metabolism in colon cancer. The IFN-γ signaling pathway plays a critical role in anticancer immunity mainly through anti-proliferation (38), apoptosis (39), necrosis (40), and ferroptosis (41); however, it is also reported that IFN-γ induces the expression of immune checkpoints which may lead to an immune-suppression microenvironment, and this was consistent with our further analysis about the immune microenvironment (42–44). Taken together, our results suggested that the IFN-γ response pathway might mainly contribute to the immune-suppression microenvironment in glycerolipid metabolism-activated colon cancer, other than antitumor effect.

The tumor immune microenvironment is critical to tumorigenesis, disease progression, and treatment outcomes in colon cancer (45, 46). Researchers generated an Immunoscore (IS) based on the density of CD3+ and CD8+ T-cell effectors within the tumor, as well as its invasive margin, and developed a colon cancer recurrence risk model accordingly (47, 48). However, due to the complexity of the immune system, all different types of immune cells could contribute to the immune microenvironment. Hence, in our study, we evaluated the infiltration of 64 cell types and compared their proportion between high- and low-GLMS groups. We observed that the infiltration proportions of B cells, macrophages, aDC, cDC, and iDC were significantly higher in the low-GLMS group; in contrast, the infiltration proportion of NKT cells was significantly higher in the high-GLMS group. It is previously reported that fatty acid metabolism, which is an important step of glycerolipid metabolism, controls the immune suppressive phenotype of tumor-associated macrophages in the colon cancer cell line as well as CRC patients (49). Dendritic cells are a group of antigen-presenting cells. Researchers have reported that accumulation of lipids, especially triacylglycerol, affects the function of dendritic cells, which leads to ineffective tumor-associated antigens presenting in the colon cancer mouse model (50). Together with this result, our analyses give us a hint that elevated DC infiltration in the low-GLMS group may be associated with glycerolipid accumulation, which might also affect the subsequent antigen-presenting process. Last but not least, the expression level of different checkpoints in the low- and high-GLMS groups showed that the expression of 12 immune checkpoints was significantly increased in the low-GLMS group which may lead to an immune-suppression microenvironment. These results supported the notion that lipid metabolism not only plays a role in the tumorigenesis and disease progression of cancer cells but also is involved in the responses of tumor recruited immune, stromal cell, and regulating immune microenvironment (21). More importantly, our single-cell transcriptomics analyses indicated that glycerolipid metabolism may affect the biological activity of malignant cells and Tprolif cells in colon cancer. It is also reported that, in hepatocellular carcinoma (HCC), palmitic acyl–containing glycerophospholipids inhibit HCC cell growth and metastatic abilities, which was consistent with our analyses (51).

There are some limitations of the study. Firstly, we developed this model based on the gene set enrichment score of the total 49 KEGG_GLYCEROLIPID_METABOLISM genes, which may cause over-interpretation about the results. Secondly, the conclusions of our study are mainly based on *in silico* bioinformatics analyses of two retrospective cohorts. The retrospective nature and sample sizes may limit the interpretation of the conclusion of the present study. Further *in vitro* or *in vivo* validation studies are needed and prospective studies are warranted.

In conclusion, we constructed an independent colon cancer prognostic factor based on the glycerolipid metabolism gene enrichment score. Our prognostic factor, GLMS, was correlated with important signaling pathways in colon cancer, as well as the immune microenvironment. In addition, GLMS mainly targeted on malignant cells in colon cancer. Thus, our findings provide a new prognostic factor for colon cancer and the possible molecular mechanism behind it.

## Data Availability Statement

The datasets generated and analyzed in the present study are available in the public data repository, TCGA-COAD: https://portal.gdc.cancer.gov/repository, and GEO: https://www.ncbi.nlm.nih.gov/geo/query/acc.cgi?acc=GSE39582.

## Author Contributions

SL and FW designed this work. ZW and ZZ integrated and analyzed the data. KZ, QZ, and SdC wrote this manuscript. HZ, GW, and SlC edited and revised the manuscript. All authors contributed to the article and approved the submitted version.

## Conflict of Interest

Authors QZ, SdC, GW, and SlC were employed by Burning Rock Biotech.

The remaining authors declare that the research was conducted in the absence of any commercial or financial relationships that could be construed as a potential conflict of interest.

## Publisher’s Note

All claims expressed in this article are solely those of the authors and do not necessarily represent those of their affiliated organizations, or those of the publisher, the editors and the reviewers. Any product that may be evaluated in this article, or claim that may be made by its manufacturer, is not guaranteed or endorsed by the publisher.
